# Prognostic Nutritional Index as a Predictor of Recurrence in Patients Undergoing Pericardiocentesis: A Retrospective Analysis

**DOI:** 10.1155/crp/5598299

**Published:** 2025-02-24

**Authors:** Ahmet Anıl Başkurt, Yusuf Demir, Oktay Şenöz

**Affiliations:** Department of Cardiology, Bakırçay University Çiğli Training and Research Hospital, Izmir, Turkey

**Keywords:** pericardial effussion, pericardiosentesis, PNI, prognostic nutritional index, tamponade

## Abstract

**Objective:** Recurrence of pericardial effusion is possible despite the successful completion of pericardiocentesis and initiation of treatment. Predicting recurrence is important for determining treatment strategies. This study aimed to examine the factors that influence the recurrence of effusion in patients who had undergone pericardiocentesis.

**Method:** A total of 113 patients with the evidence of tamponade or pericardial effusion over 10 mm were included in the study. The mean follow-up period was 49 months. Patients with and without recurrent effusion were divided into two groups. PNI calculation (PNI = 10 × serum albumin (g/dL) + 0.005 × total lymphocyte count (mm^3^) formula was used.

**Results:** Recurrent pericardial effusion was observed in 30 patients during the follow-up period. There was no difference in age, gender, hypertension, LVEF%, hypertension, and appearance of fluid when the two groups were compared. There was a difference in PNI score and presence of malignancy between the two groups (*p*: 0.031 and 0.042, respectively). Multivariate logistic regression showed that malignancy and PNI score were independent predictors of recurrence in patients undergoing pericardiocentesis (*p*: 0.015 and *p*: 0.014, respectively). In the ROC analysis, PNI < 40.75 predicts recurrent pericardial effusion with 75% sensitivity and 58% specificity (AUC: 0.626, 95% CI: 0.509–0.742, and *p*=0.042).

**Conclusion:** Predictors of recurrence in patients undergoing pericardiocentesis are important for patient follow-up. PNI is a simple and useful score that can be used to predict recurrent pericardial effusion.

## 1. Introduction

Pericardial effusion is the buildup of fluid in the sac surrounding the heart, and it can occur in people of all ages and populations. There is a lack of data regarding the incidence and prevalence of pericardial effusions [1]. The main challenge for the physician is to try to determine the cause of the condition. The main cause of effusion varies depending on demographic variables such as geographic location, age, and presence of additional illnesses [2, 3]. Pericardial effusions are most commonly caused by infections (such as viral, bacterial, and mainly tuberculosis), malignancies, connective tissue illnesses, and myocardial damage syndromes (such as effusions following a cardiac event or after cardiac surgery) [4]. Effusion can be categorized as transudative, exudative, or sanguinous [5]. The signs and symptoms of pericardial effusion differs depending on the speed at which fluid accumulates in the pericardium. Typical symptoms include difficulty breathing during physical activity that worsens when lying down, chest pain, and/or an unpleasant feeling of being full. Pericardial effusion can manifest with a wide spectrum of clinical presentations, ranging from a medically unimportant, coincidental discovery to a potentially fatal condition known as cardiac tamponade. The significant range in measurements is primarily attributed to the fluctuating rate at which pericardial fluid accumulates. Even a little amount of 100 mL of fluid might produce reduced ventricular filling and decreased cardiac output in cases of acute accumulation. On the other hand, chronic and slow accumulation can result in substantial effusions of one to two liters without causing major hemodynamic consequences [6]. Echocardiography is mostly used to diagnose pericardial effusion, and it additionally facilitates a semiquantitative examination of the degree and hemodynamic impact of the effusion [7]. The management of pericardial effusion should primarily focus on treating the underlying cause [8]. Pericardiocentesis is essential for both the diagnosis and management of these patients. Recurrence of pericardial effusion is possible despite the successful completion of pericardiocentesis and initiation of treatment. Predicting recurrence is important for determining treatment strategies. This study aimed to examine the factors that influence the recurrence of effusion in patients who had undergone pericardiocentesis.

## 2. Procedure

The puncture site is 1 cm left of the costoxiphoid angle and 1 cm below the costal arch. An 18G puncture needle is inserted from the determined point, making a 30–45 degree angle with the abdominal wall and pointing towards the left shoulder. Meanwhile, the path of the needle from the apical four-chamber view to the right ventricle and atrium is monitored by echocardiography. During aspiration, if fluid comes into the syringe, the pericardial space has been entered. A soft J-tipped guide wire is advanced through the needle into the pericardial cavity and the needle is removed by leaving the wire in place. After cutting the skin with a scalpel, a 6F dilatator is advanced over the wire to expand the subcutaneous and pericardial space. Then, a multihole soft pigtail catheter is inserted through the wire into the pericardial cavity. The dilator and catheter should not be placed unless it is certain that the pericardial cavity has been entered.

## 3. Patient and Method

A total of 113 patients who underwent pericardiocentesis between 01/01/2016 and 01/01/2024 were retrospectively included in the study. Patients who did not undergo pericardiocentesis despite having pericardial fluid were excluded. A total of 113 patients with evidence of tamponade or pericardial effusion over 10 mm were included in the study. The mean follow-up period was 49 months. During this period, recurrent pericardial effusion was observed in 30 patients. Patients with and without recurrent effusion were divided into two groups. Samples obtained from the first pericardiocentesis procedure and simultaneously obtained blood parameters were analyzed. Procedure techniques and comorbidities of the patients were analyzed from the hospital record system. PNI calculation (PNI = 10 × serum albumin (g/dL) + 0.005 × total lymphocyte count (mm^3^) formula was used. COVID-19 infection patients were excluded in the study.

## 4. Statistical Analysis

Data were evaluated in SPSS 22.0 (IBM Corporation, Armonk, NY, USA) programme. The normal distribution of variables was evaluated with the Kolmogorov–Smirnov test, and the homogeneity of variance was evaluated with the Levene test and all continuous variables presented normal distribution. Data determined by measurement were given as the mean and standard deviation for those with normal distribution. The unpaired *t*-test was used in the statistical analysis of these data. Categorical data were shown as absolute and relative frequencies and in the statistical analysis, the χ^2^ test or Fisher's exact test was used, as appropriate. Variables were analyzed at a 95% confidence interval, and *p* < 0.05 was considered as significant. Univariate logistic regression and multivariate logistic regression were used to identify independent predictors. Receiver operating characteristic (ROC) analysis was used for area under the curve (AUC) calculations.

## 5. Results

The mean age of patients who underwent pericardiocentesis was 61.85 ± 16.60 years and 61.9% were male. Prognostic nutrition index (PNI) scores of the patients mean 46.16 ± 9.64 determined. A total of 54.9% of the patients had hypertension, 22.1% had diabetes mellitus, and 16.8% had chronic renal failure. Pericardiocentesis was performed in 88.5% of 113 patients with evidence of tamponade. The effusion samples were 91.2% exudative, 8.8% transudative, 61.1% haemorrhagic, and 38.9% serous. A total of 30 of 113 patients had recurrent pericardial effusion. A total of 8 of the 30 patients with recurrence came in the tamponade clinic, and 22 of them came back due to dyspnea, chest pain, etc. accompanied by massive pericardial effusion. There was no difference in age, gender, hypertension, LVEF%, hypertension, and appearance of fluid when patients with and without recurrent pericardiocentesis were compared. There was a difference in the PNI score and presence of malignancy between the two groups (*p*: 0.031 and 0.042, respectively) (Table 1).

Analyses of blood and pericardial samples in patients with recurrent and nonrecurrent pericardial effusions are given in Table 2.

We performed univariate logistic regression for age, the contents of the fluid, malignancy, and PNI score with *p* values below 0.1 and found *p* values of 0.067, 0.091, 0.045, and 0.041, respectively. Subsequent multivariate logistic regression showed that malignancy and PNI score were independent predictors of recurrence in patients undergoing pericardiocentesis (*p*: 0.015 and *p*: 0.014, respectively) (Table 3).

**Figure 1:**
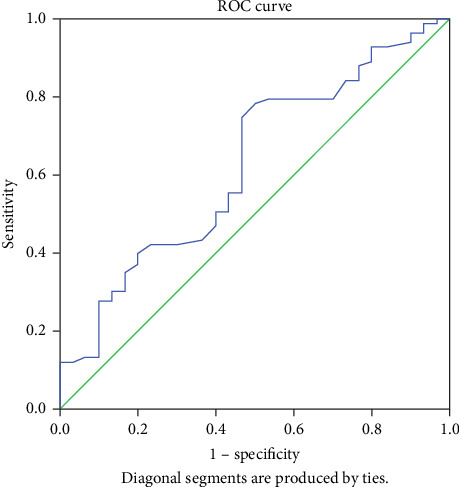
Receiver operating characteristics curve of prognostic nutritional index (PNI) for predicting development of recurrent pericadial effusion.

In the ROC analysis (Figure 1), PNI < 40.75 predicts recurrent pericardial effusion with 75% sensitivity and 58% specificity (AUC: 0.626, 95% CI: 0.509–0.742, and *p*=0.042).

## 6. Discussion

Low PNI score and presence of malignancy predict recurrence of pericardial effusion. In the presence of pericardial fluid, the patient's hemodynamics should be evaluated and urgent pericardiocentesis may be required according to the presence of tamponade. If there are no signs of tamponade, diagnostic or therapeutic pericardiocentesis may be performed. The 2015 guidelines from the European Society of Cardiology (ESC) recommended drainage of fluid to the pleura or peritoneum by pericardial window opening or percutaneous pericardial balloon methods in patients who underwent recurrent pericardiocentesis and did not respond to medical treatment [9].

The etiology of pericardial fluid should be determined and treatment specific to the underlying pathology should be initiated. Even though pericardiocentesis successfully reduces symptoms and enhances blood flow, fluid builds again in as many as 60% of the cases [10, 11]. A thorough review of 31 nonrandomised studies on percutaneous interventions for malignant pericardial effusion found that procedures other than isolated pericardiocentesis were linked to much lower rates of recurrence [12].

When pericardial effusions last for weeks, months or even longer, they are called chronic effusion or pericarditis with chronic effusion. In these situations, diagnostic tests generally do not show the reason, but over time, an etiology may become clear.

A study looked at 100 patients with unknown chronic large pericardial effusions who were brought to one of three Italian referral centers between 2000 and 2015. During the follow-up (mean 50 months), only eight of them developed cardiac tamponade. Only a very small number of cases (28 or 2.5%), out of 1108 people who first got pericarditis between 1977 and 1992, had a big idiopathic effusion that lasted for an average of three years [13]. About 58% of the patients in this study got cardiac tamponade at some point during the follow-up (median: three years).

Patients should be followed up after pericardiocentesis because unexpected signs of tamponade may develop. Clinicians require a biomarker that is clinically pertinent, economical and straightforward to interpret in order to assist in the prediction of recurrence. One of the most important predictors of recurrent pericardial effusion is the presence of malignancy. In our study, 47% of the patients who underwent recurrent pericardiocentesis had malignancy. Another finding was that the PNI score was found to be high in patients with recurrent pericardial effusion. Although the PNI score was low in patients with malignancy in the study, regression analysis showed that a low PNI score predicted recurrence independently of malignancy. PNI is a combined marker that reflects both the immunological and inflammatory status and the nutritional status of the individual [14]. In 1984, Japanese scientists Onodera, Goseki, and Kosaki [15] initiated the assessment of the nutritional and immunological status of cancer patients undergoing gastrointestinal surgery. PNI has become recognized as a novel marker for the prognosis of numerous diseases, including diabetic nephropathy, heart failure, and COVID-19, in recent years [16–23]. However, the correlation between pericardiocentesis patients and PNI remains unclear and there are no studies in the literature.

The primary mechanism by which hypoalbuminemia increases cardiovascular risk is the diminished antioxidant, oncotic pressure-maintaining, and antithrombotic capacities of albumin [24]. Impaired immune defenses as a result of malnutrition are indicated by decreased absolute lymphocyte counts [25]. In pericardial effusion, inflammation occurs in the pericardium. Low PNI score seems to be a parameter, indicating this inflammation and predicting recurrence. In our study, we found that in addition to malignancy, low PNI score was an independent predictor of recurrence in patients undergoing pericardiocentesis.

In a study published in recent years, it has been shown that opening a pericardial window reduces recurrences without making a mortality difference [26]. In our study, 8 of the 30 patients with recurrence came in the tamponade clinic, 22 of them came back due to dyspnea, chest pain, etc. accompanied by massive pericardial effusion. Our recommendation in daily practice is to follow up more strictly in patients with low PNI score and to be more alert in terms of recurrence. In centers with experienced surgeons, the decision of pericardiocentesis or pericardial window opening in patients with low PNI score or malignancy should be made by the heart team.

## 7. Conclusion

Predictors of recurrence in patients undergoing pericardiocentesis are important for patient follow-up. PNI is a simple and useful score that can be used to predict recurrent pericardial effusion.

## Figures and Tables

**Table 1:** Data of recurrent and nonrecurrent pericadial effusion patients.

Variables	Recurrent patients *n*: 30	Nonrecurrent patients *n*: 83	*p* value
Age, years, mean ± sd	57.03 ± 17.01	63.59 ± 16.20	0.063
Sex, male, *n* (%)	19 (63%)	51 (61%)	0.855
PNI score, mean ± sd	43.03 ± 8.77	47.29 ± 9.75	**0.031**
Hypertension, *n* (%)	16 (53%)	46 (55%)	0.844
Diabetes mellitus, *n* (%)	5 (17%)	20 (24%)	0.401
Chronic kidney disease, *n* (%)	6 (20%)	13 (16%)	0.586
Heart failure, *n* (%)	1 (0.3%)	8 (0.9%)	0.441
The contents of the fluid, transudate, *n* (%)	5 (17%)	5 (0.6%)	0.127
Appearance of the liquid, haemorrhagic, *n* (%)	15 (50%)	54 (635)	0.079
Tamponade, *n* (%)	28 (93%)	72 (87%)	0.333
Malignancy, *n* (%)	14 (47%)	22 (27%)	**0.042**
LVEF%, mean ± sd	59.78% ± 1.04	58.53% ± 5.50	0.123

**Table 2:** Analysis of blood and pericardial samples in patients with recurrent and nonrecurrent pericardial effusion.

Variables	Recurrent patients *n*: 30	Nonrecurrent patients *n*: 83	*p* value
Pericardial fluid glucose, mean ± sd	67.50 ± 43.44	135.50 ± 119.61	0.299
Pericardial fluid LDH, mean ± sd	873.50 ± 969.05	794.12 ± 1005.94	0.896
Pericardial fluid albumin, mean ± sd	29.20 ± 9.88	30.16 ± 9.55	0.872
Pericardial fluid triglycerides, mean ± sd	47.48 ± 54.21	54.23 ± 31.96	0.788
Blood sample albumin, mean ± sd	3.64 ± 0.68	3.93 ± 0.78	0.073
Blood sample glucose, mean ± sd	115.72 ± 26.41	115.54 ± 28.88	0.976
Blood sample hemoglobulin, mean ± sd	12.80 ± 1.87	12.75 ± 1.81	0.900
Blood sample neuthrophil, mean ± sd	5406.67 ± 2281.40	5052.05 ± 1462.28	0.334
Albumin gradient, mean ± sd	0.79 ± 0.42	0.68 ± 0.37	0.167

**Table 3:** Univariate and multivariate logistic regression analysis results.

Variables	Univariate logistic regression OR (%95 CI)	*p* value	Multivariate logistic regression OR (%95 CI)	*p* value
Age	0.977 (0.952–1.002)	**0.067**	0.972 (0.944–1.001)	0.061
The contents of the fluid	3.120 (0.834–11.667)	**0.091**	3330 (0.729–15.206)	0.120
Malignancy	1.248 (1.005–1.550)	**0.045**	1352 (1.061–1.722)	**0.015**
PNI score	0.951 (0.907–0.998)	**0.041**	0.940 (0.894–0.987)	**0.014**

## Data Availability

Data used to support the findings of this study are available from the corresponding author upon reasonable request.
